# Comparison of respiratory-gated and breath‑hold accelerated T2-weighted sequences for liver MRI with deep learning reconstruction

**DOI:** 10.1186/s41747-026-00679-1

**Published:** 2026-02-23

**Authors:** Hualing Li, Chenglin Hu, Qiuxia Wang, Yan Luo, Gen Chen, Xuemei Hu, Xiaopeng Song, Runyu Tang, Qiufeng Liu, Yang Yang, Zhen Li

**Affiliations:** 1 Department of Radiology, Tongji Hospital, Tongji Medical College, Huazhong University of Science and Technology, Wuhan, China; 2 Central Research Institute, United Imaging Healthcare, Shanghai, China; 3 Medical School, Faculty of Medicine, Tianjin University, Tianjin, China

**Keywords:** Artifacts, Deep learning, Liver, Magnetic resonance imaging, Respiration

## Abstract

**Background:**

T2-weighted imaging (T2WI) of the liver suffers from prolonged scan times and respiratory motion artifacts. Deep learning (DL)-based reconstruction can accelerate acquisition while maintaining diagnostic quality. We compared respiratory-gated (RG) and breath-hold (BH) DL-T2WI to radial k-space sampling acquisition and reconstruction with motion suppression (ARMS)-T2WI and evaluated how respiratory characteristics affect image quality.

**Materials and methods:**

We prospectively enrolled 120 participants who underwent 3-T RG DL-, BH DL-, and ARMS-T2WI. Three radiologists evaluated image quality and lesion conspicuity using a 5-point scale. Respiratory characteristics were extracted from breathing curves.

**Results:**

All sequences showed comparable lesion-to-liver contrast ratios (*p* = 0.139), detection rates (*p* = 0.106), and lesion conspicuity scores (*p* = 0.990). RG DL-T2WI showed higher overall image quality compared to BH DL-T2WI (*p* = 0.027), and similar scores to ARMS-T2WI (*p* = 0.106). A respiratory score calculated using four parameters predicted ARMS-T2WI image quality with an area under the receiver operating characteristic curve (AUROC) of 0.836 (95% confidence interval 0.638–0.968) in the validation set. For RG DL-T2WI, a respiratory score using seven parameters achieved an AUROC of 0.831 (0.652–0.967) in the validation set. Standard deviation of the respiratory amplitude (SD_amp_) was an independent factor for BH DL-T2WI image quality (validation set, odds ratio 0.297, *p* = 0.049). For patients with high SD_amp_, RG DL-T2WI provided better image quality compared to BH DL-T2WI (68.6% *versus* 14.3%, *p* < 0.001).

**Conclusion:**

Both RG and BH DL-T2WI offer image quality comparable to ARMS-T2WI. Respiratory metrics derived from breathing curves may facilitate personalized liver imaging.

**Relevance statement:**

Both respiratory-gated and breath-hold T2WI with deep learning reconstruction showed comparable image quality to T2WI based on radial k-space sampling strategies. Respiratory parameters enable personalized magnetic resonance liver imaging workflows.

**Key Points:**

- Respiratory-gated and breath-hold deep learning T2WI exhibited satisfactory image quality.
- Respiratory curve traits variably impact T2WI quality, guiding personalized imaging workflows.‌
- Respiratory-gated deep learning-reconstructed T2WI benefits patients with breath-holding difficulties in liver MRI.

**Graphical Abstract:**

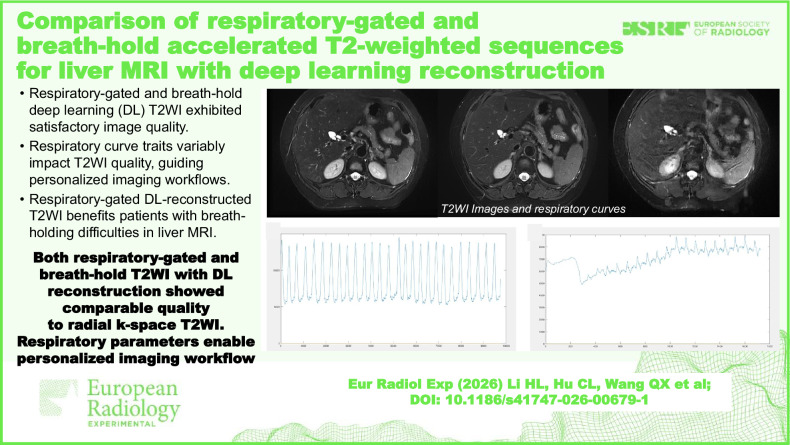

## Background

Magnetic resonance imaging (MRI) is a superior liver imaging modality compared to ultrasonography and computed tomography, offering multiparametric information without exposing patients to ionizing radiation [1, 2]. T2-weighted imaging (T2WI) is a crucial component of liver MRI protocols, essential for detecting and characterizing hepatic lesions [3, 4]. However, liver T2WI often involves prolonged acquisition times and is susceptible to motion artifacts caused by patient respiration.

Advanced fast spin-echo (FSE) sequences based on sophisticated k-space sampling strategies (*e.g*., PROPELLER by GE Healthcare, BLADE by Siemens Healthineers, ARMS by United Imaging Healthcare) have significantly reduced artifacts and enhanced image quality [5–7]. However, the unique k-space sampling patterns result in prolonged acquisition times [5]. When combined with respiratory-gating technology, which triggers image acquisition with specific phases of the respiratory cycle, acquisition times are further extended, particularly for patients with shallow or irregular breathing patterns [4, 7].

A recent approach integrates deep learning (DL) reconstruction to achieve higher acceleration factors while concurrently enhancing image quality [8–10]. Previous studies have demonstrated that DL reconstruction, combined with the FSE sequence, can complete the T2WI sequence within 1 to 2 breath-holds [11–13]. This technique has shown comparable image quality and diagnostic confidence to conventional FSE sequences [11–13]. However, not all patients can comply with breath-holding, particularly those with abdominal pain, hearing impairment, children, or advanced age [14, 15]. The respiratory-gated DL-FSE sequence allows data acquisition during free breathing and significantly reduces scan time. Despite its potential, its clinical efficacy still requires further validation.

Furthermore, motion artifacts caused by respiratory movements are the most common reason for repeating liver MRI sequences [15, 16]. Variations in respiratory characteristics among individuals are very common. Respiratory characteristics are influenced by numerous factors such as physical activity, body position, sex, age, emotion, and health conditions [17–19]. However, the direct relationship between respiratory curve characteristics and the image quality of T2WI for liver remains unclear. Different T2WI sequences for liver MRI utilize different strategies to reduce respiratory motion artifacts [4, 11]. For example, ARMS-T2WI employs radial acquisitions from the center of k-space to reduce artifacts [5, 7]. In contrast, DL-T2WI utilizes artificial intelligence (AI)-driven random k-space sampling and reconstruction algorithms, enabling faster scans with fewer artifacts [11, 20–22]. Evaluating and comparing the efficacy of different T2WI protocols across various patient types and their respiratory characteristics is crucial for optimizing liver MRI scan protocols in clinical practice.

Therefore, this prospective study aims to compare respiratory-gating and breath-hold DL-T2WI sequences with ARMS-T2WI (radial k-space sampling strategies) for liver MRI at 3 T. Additionally, it seeks to explore the impact of patients’ respiratory characteristics on image quality across different T2WI protocols to enable personalized scanning protocols.

## Materials and methods

### Study population

**Fig. 1:**
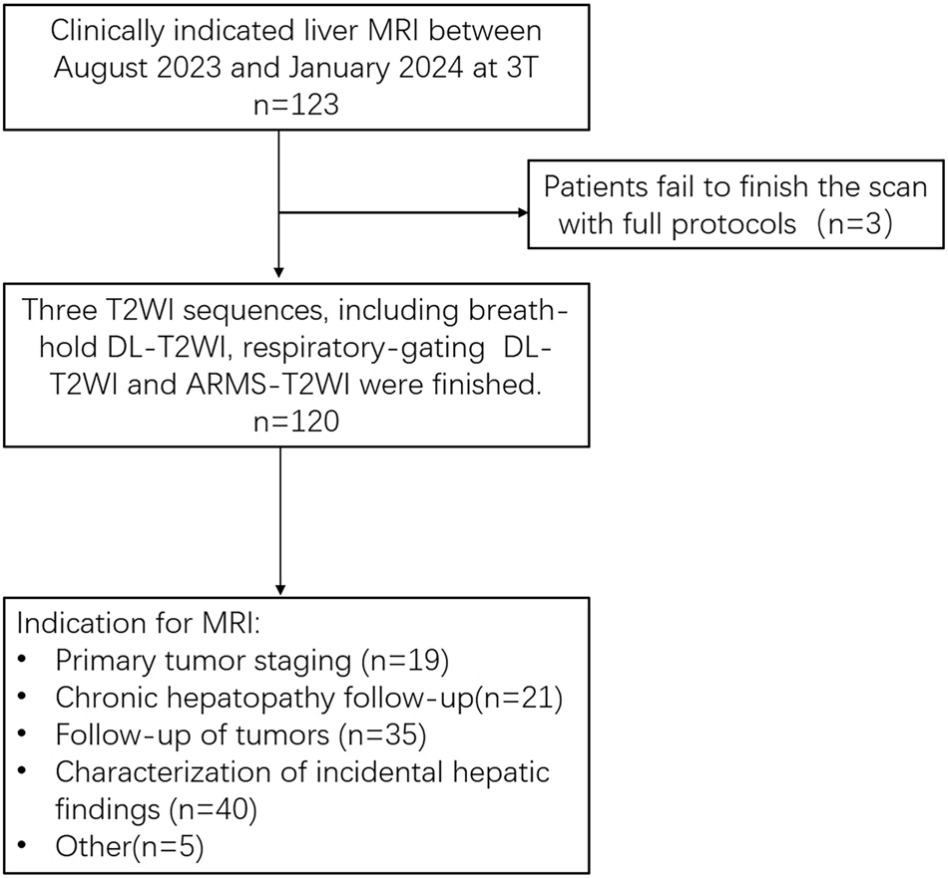
Flow chart of the study population

This prospective study was approved by the review board of our institute (TJ-IRB20230818) on August 15, 2023, and all participants provided written informed consent. Consecutive adult patients referred to our institution for clinically indicated liver MRI between August 2023 and January 2024 were included in this study (Fig. 1). A total of 120 patients were finally included.

### MRI acquisitions

All MRI examinations were conducted using a 3-T scanner (uMR 790; United Imaging Healthcare) equipped with a 24-channel body array and a 32-channel spine array. A wireless respiratory sensor, featuring a pressure transducer, was positioned above the midpoint between the xiphoid process and the navel of each participant, securely fastened with a belt. Before the examination, standard respiratory training tailored to each patient was routinely administered.

The protocol for liver examination in this study comprised the following sequences:

1. standard axial T2-weighted FSE with fat suppression sequences, *i.e.*, RG acquisition and reconstruction with motion suppression (ARMS)-T2WI, RG DL-T2WI, and single breath-hold (BH) DL-T2WI, performed in a random order;
2. coronal single-shot FSE with fat suppression;
3. diffusion-weighted imaging; and
4. dynamic imaging with 0.1 mmol/kg of gadobenate dimeglumine (Multihance, Bracco Imaging) injected via a high-pressure injector (2.5 mL/s).

Detailed acquisition parameters are summarized in Table 1.

**Table 1:** Acquisition parameters of the T2WI sequences

Parameters	RG AMRS-T2WI	RG DL-T2WI	BH DL-T2WI
Respiration	Respiratory-gated	Respiratory-gated	Single breath-hold
TR, ms	3,883	3,352	3,381
TE, ms	83.84	84	84
Slice thickness, mm	5	5	5
Slice gap, mm	1	1	1
Matrix size	304*304	212*288	212*288
FOV	400*400	280*380	280*380
Voxel size, mm^3^	1.32*1.32*5	1.32*1.32*5	1.32*1.32*5
Echo train length	31	61	61
Acceleration	2.58	3.48	3
TA, s	222.5 ± 47.3	31.9 ± 8.4	17

*ARMS* reconstruction with motion suppression, *BH* breath-hold, *DL* deep learning, *FOV* field of view, *RG* respiratory-gated, *TA* acquisition time, *TE* echo time, *TR* repetition time

### DL image reconstruction

Images were reconstructed inline using a commercial DL pipeline (United Imaging Healthcare, uMR 790), which is USA Food and Drug Administration‒FDA 510(k)-cleared for clinical use. The DL algorithm was trained using a dataset of two million fully sampled images, comprising 2% phantoms and 98% human volunteers. The primary network architecture was an extended fully convolutional neural network based on U-net, featuring residual blocks with skip connections to improve learning efficiency [11]. Full k-space data were retrospectively undersampled and transformed into image space to serve as model inputs, while the corresponding images reconstructed from the full k-space data were used as target outputs [23]. The mathematical model integrated components of compressed sensing, partial Fourier, and parallel imaging, with a regularization term added to the reconstruction equation to ensure alignment between reconstructed images and the fully sampled reference standard.

### Image analysis

Three radiologists (Reader1, Reader2, and Reader3 with 7, 9, and 15 years of experience in abdominal imaging, respectively) evaluated the image quality of the three sequences independently and randomly. The readers were blinded to the acquisition method and patients’ characteristics. The time interval between sequence assessments was at least 30 days. Overall image quality, artifacts, sharpness of liver margin, sharpness of intrahepatic vessel margin, the conspicuity of the main lesion, and cardiac motion-related signal loss (CRSL) were evaluated on an ordinal 5-point Likert scale. Disagreements were resolved through discussion and consensus. The detailed image quality evaluation criteria are shown in Table S1. Good image quality was considered when the overall image quality score reached 4 or higher [24–26].

For each sequence, the total number of focal hepatic lesions and the size of visible lesions were recorded independently by Reader1 and Reader2. Lesions < 5 mm in diameter were excluded from diameter measurement. The locations of lesions were recorded according to the Couinaud segments [27]. To establish a standardized reference for lesion detection, an experienced radiologist (Reader3, with 15 years of experience in abdominal imaging) reviewed all sequences, along with clinical and laboratory data, to ascertain lesion location and number. A false-positive lesion was defined as a candidate without a corresponding lesion on the reference standard, which was established by: (1) histopathology; (2) multiparametric MRI confirming lesion absence; or (3) negative imaging follow-up ≥ 6‒12 months. Ambiguous or non-lesion exclusions (not counted as true positives or false positives) were: (1) motion or respiratory ghosting mimicking focal hyperintensity; (2) peribiliary cysts or ductal fluid collections misinterpreted as parenchymal nodules; (3) vessel-related partial-volume or slab boundary hyperintensity; and (4) ill-defined subcapsular edema. Representative examples are provided in Supplementary Fig. S1.

Quantitative assessment of the three sequences was performed by Reader1 and Reader2 independently. Liver parenchymal regions of interest (ROIs) measuring 100 mm^2^ were positioned on the lateral, medial, anterior, and posterior segments of the liver, avoiding blood vessels and artifacts. In modern parallel imaging and DL reconstructions, noise is spatially varying and non-Gaussian; therefore, the signal-to-noise ratio (SNR) derived from a background ROI in air was considered an apparent SNR and is interpreted with caution in this study [28]. For assessing the noise level, the ROI was manually positioned in the right portion of the background air. The lesion ROIs were manually delineated to include the whole lesions on the largest cross-section of the lesion. The signal intensity (SI) of each ROI was documented for each reader. The liver apparent signal-to-noise ratio (SNR_app_) and the contrast ratio (CR) between lesions and liver parenchyma were assessed by two readers using the following equations:

$${{{{\rm{SNR}}}}}_{{{{\rm{app}}}}}={{{{\rm{SI}}}}}_{{{{\rm{liver}}}}}/{{{\rm{SD}}}}$$

$${{{\rm{CR}}}}=({{{{\rm{SI}}}}}_{{{{\rm{lesion}}}}}\,{minus}\,{{{{\rm{SI}}}}}_{{{{\rm{liver}}}}})/({{{{\rm{SI}}}}}_{{{{\rm{lesion}}}}}\,{plus}\,{{{{\rm{S}}}}{{{\rm{I}}}}}_{{{{\rm{liver}}}}})$$

SI_liver_ and SI_lesion_ represent the mean signal intensities of the liver parenchyma and each lesion, respectively, while SD denotes the standard deviation of the noise.

### Respiratory curve

**Fig. 2:**
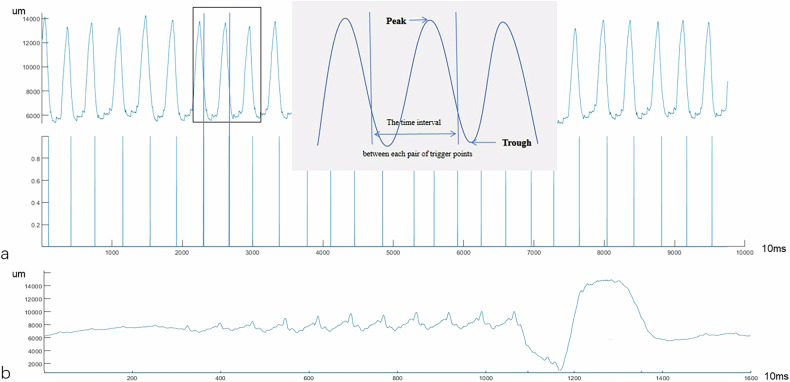
Schematic diagram of respiratory curve parameters. **a** Respiratory curve and trigger point for respiratory-gated T2WI sequence. **b** Respiratory curve for breath-hold T2WI sequence. Fluctuations were observed during the final phase of scanning

The original breathing curve data were recorded with the sensor belt, with time on the *x*-axis and respiration amplitude on the *y*-axis in the plots (Fig. 2). Trigger points, defined by a signal drop of 35% from the baseline, were recorded and marked on the curve.

For the respiration-gated acquisition sequences, the following parameters were calculated [29]: The average and standard deviation of the peaks (AVG_amp_ peak, SD_amp_ peak) and troughs (AVG_amp_ trough, SD_amp_ trough) in the breathing curve, the average (AVG_breath-time_) and standard deviation (SD_breath-time_) of the time intervals between each pair of trigger points, and the average and standard deviation of the respiratory amplitude at the trigger points (AVG_amp_ trigger point, SD_amp_ trigger point). The coefficient of variation of breathing rate was also calculated.

For BH DL-T2WI, the following parameters were collected: average value and standard deviation of the respiratory amplitude (BH-AVG_amp_ and BH-SD_amp_, respectively).

### Construction and validation of the respiratory signature

To minimize the potential influence of the scanning sequence on patient respiration, respiratory parameters were compared between the two respiratory-gated sequences using the Wilcoxon signed-rank test. Consistent respiratory parameters across both sequences for each patient were then used for subsequent analysis.

Patients were randomly assigned to the training (*n* = 84) and validation sets (*n* = 36) in a ratio of 7:3. Taking into account the multicollinearity among respiratory parameters, the least absolute shrinkage and selection operator (LASSO) model was applied separately to each sequence to identify the respiratory parameters most strongly associated with image quality in the training set. To enhance model generalizability, hyperparameter optimization was conducted using 10-fold cross-validation prior to final model development. Using the finalized models, respiratory scores for each patient in the training and validation sets were calculated separately for the two sequences.

For BH DL-T2WI, given the limited number of respiratory parameters, multivariable logistic regression analyses were performed to assess their impact on overall image quality.

Predicting performances were quantified using the area under the receiver operating characteristic curve (AUROC). Cutoffs were determined by maximizing the Youden index based on the training set. To further assess model robustness and clinical utility, calibration curves, bootstrap-corrected AUROCs, and decision-curve analyses were also conducted.

### Statistical analysis

The differences in quantitative and subjective evaluation of image quality among the three T2WI sequences were assessed using the Kruskal–Wallis test. Pairwise differences between groups were analyzed using a post hoc Wilcoxon signed-rank test with Bonferroni correction. Spearman correlation coefficients were calculated to explore the association between the image quality scores and breathing curve features. A ρ value of less than 0.20 indicated a poor correlation; 0.21‒0.50, a fair correlation; 0.51‒0.70, a moderate correlation; 0.71‒0.90, a strong correlation; and 0.91‒1.00, a perfect correlation [30]. To assess inter-observer agreement, we calculated the intraclass correlation coefficient (ICC), a reliability statistic that quantifies the consistency of measurements made by different observers on the same subjects; ICC values > 0.75 were regarded as indicating good agreement [31]. Statistical analysis was performed with SPSS (version 23.0, Statistical Package for the Social Sciences, IBM) and R software (version 4.0.3, R Project for Statistical Computing, www.r-project.org). All *p*-values were two-sided. *p* < 0.05 was considered statistically significant.

## Results

### Patient characteristics

A total of 120 study participants were analyzed in this study. The mean age ± SD was 52.2 ± 13.6 years, and 74 participants (61.7%) were men.

### Acquisition time

The acquisition time for the RG DL-T2WI sequence was 31.9 ± 8.4 s (range, 17‒69 s). This was significantly shorter than the acquisition time for RG ARMS-T2WI (*p* < 0.001), which was 222.5 ± 47.3 s (range, 123‒395 s). The acquisition time for the BH DL-T2WI sequence was 17 s (single BH).

### Assessment of image quality

The SNR_app_ values of the liver parenchyma were significantly better for the RG ARMS-T2WI than for the RG DL-T2WI and BH DL-T2WI sequence (*p* < 0.001; Table 2). All three sequences demonstrated comparable contrast ratios (CR) for focal liver lesions relative to the adjacent liver (*p* = 0.139; Table 2).

**Table 2:** The quantitative analysis of the respiratory-gated (RG) ARMS-T2WI, RG deep learning (DL)-T2WI, and breath-hold DL-T2WI sequences

	Measured value (mean ± SD)			*p*-value^a^	*p*-value^b^		
	ARMS	RG DL-T2WI	BH DL-T2WI		ARMS *versus* RG DL-T2WI	ARMS *versus* BH DL-T2WI	RG DL-T2WI *versus* BH DL-T2WI
SNR_app_	199.15 ± 46.4	118.84 ± 43.08	109.26 ± 39.64	< 0.001	< 0.001	< 0.001	0.279
CR	0.45 ± 0.22	0.44 ± 0.23	0.51 ± 0.24	0.139			

*ARMS* reconstruction with motion suppression, *BH* breath-hold, *CR* contrast ratio between lesions and liver parenchyma, *DL* deep learning, *SNR*_*app*_ apparent signal-to-noise ratio, *RG* respiratory-gated

^a^* p*-value of Kruskal–Wallis test

^b^ *p*-value of a post hoc Wilcoxon signed-rank test with the Bonferroni correction

**Fig. 3:**
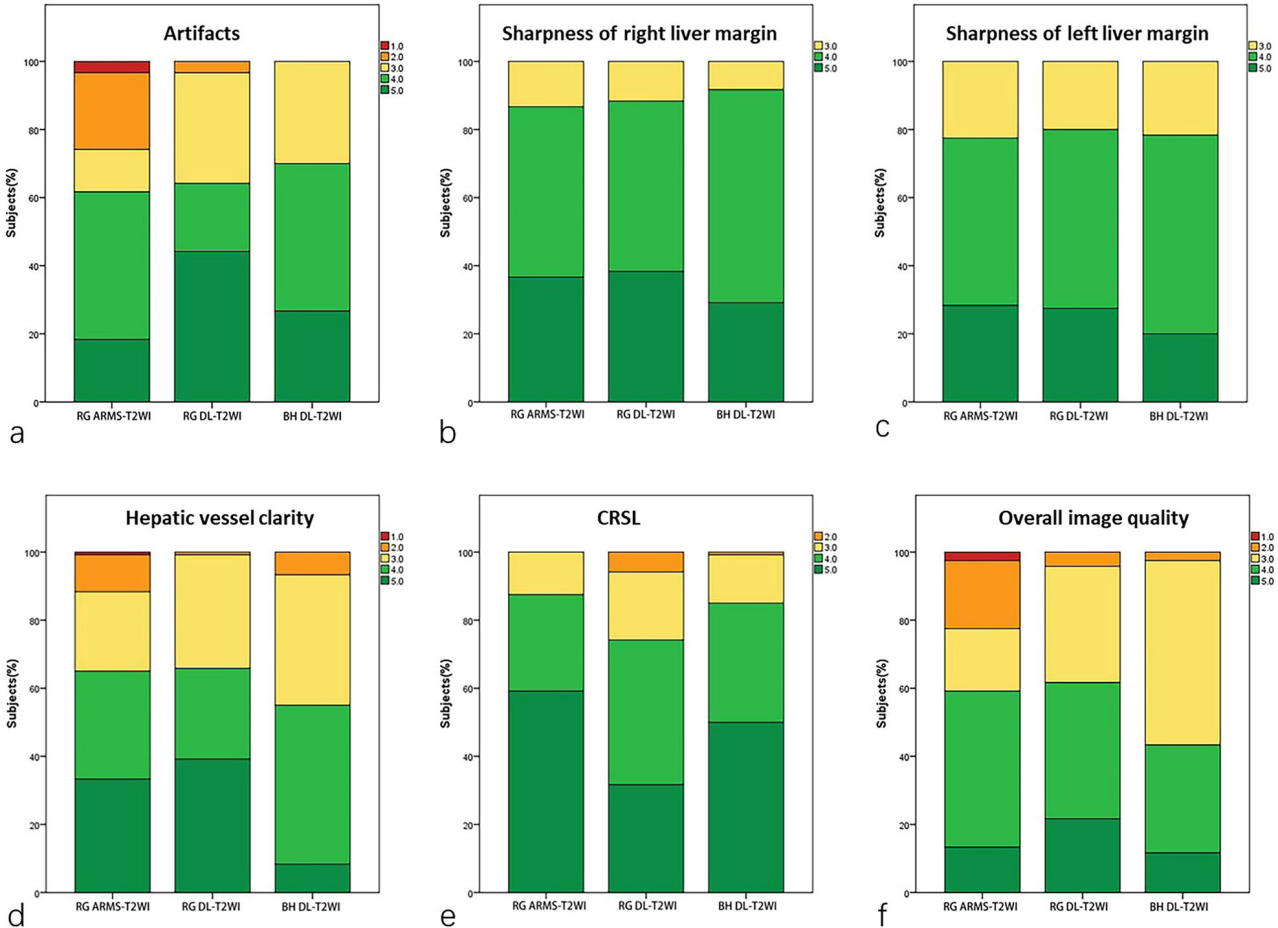
Bar plots show distributions of artifacts (**a**), sharpness of liver margin (**b**, **c**), hepatic vessel clarity (**d**), cardiac motion-related signal loss (CRSL) (**e**), and overall image quality (**f**) of respiratory-gated (RG) ARMS-T2WI, RG deep learning (DL)-T2WI, and breath-hold DL-T2WI sequences

The results of the subjective assessment of image quality for the RG DL-T2WI, BH DL-T2WI and RG ARMS-T2WI sequences are summarized in Table 3 and Fig. 3. RG DL-T2WI and RG ARMS-T2WI exhibited significantly higher scores for the sharpness of intrahepatic vessel margins compared to BH DL-T2WI (*p* < 0.001). RG ARMS-T2WI showed more severe motion artifacts than RG DL-T2WI and BH DL-T2WI (*p* = 0.001). RG ARMS-T2WI and BH DL-T2WI exhibited less severe CRSL and were rated significantly higher than RG DL-T2WI (*p* = 0.001). RG DL-T2WI demonstrated similar overall image quality scores compared to RG ARMS-T2WI (*p* = 0.106) but scored higher than BH DL-T2WI (*p* = 0.027). The overall image quality score was 4 or higher for 43.3% (52/120) of patients with BH DL-T2WI, which was significantly lower than RG DL-T2WI (61.7%, 74/120) and RG ARMS-T2WI (59.2%, 71/120) (*p* = 0.008).

**Table 3:** Subjective assessment of image quality for the respiratory-gated (RG) ARMS-T2WI, RG deep learning (DL)-T2WI, and breath-hold DL-T2WI sequences

	Measured value (Mean ± SD)			*p*-value^a^	*p*-value^b^		
	ARMS	RG DL-T2WI	BH DL-T2WI		ARMS *versus* RG	ARMS *versus* BH	RG *versus* BH
Artifacts	3.51 ± 1.13	4.05 ± 0.95	3.97 ± 0.76	0.001	0.001	0.019	1
Sharpness of the left liver margin	4.23 ± 0.67	4.27 ± 0.66	4.21 ± 0.58	0.668	-	-	-
Sharpness of the right liver margin	4.06 ± 0.71	4.08 ± 0.69	3.98 ± 0.65	0.529	-	-	-
Hepatic vessel clarity	3.86 ± 1.03	4.04 ± 0.87	3.57 ± 0.74	< 0.001	0.725	0.013	< 0.001
CRSL	4.47 ± 0.71	4.00 ± 0.87	4.34 ± 0.75	< 0.001	< 0.001	0.578	0.005
Overall image quality	3.48 ± 1.04	3.79 ± 0.83	3.53 ± 0.73	0.021	0.106	1	0.027
Lesion conspicuity	4.16 ± 0.92	4.35 ± 0.85	4.32 ± 0.84	0.990			1

*ARMS* reconstruction with motion suppression, *BH* breath-hold, *CRSL* cardiac motion-related signal loss, *DL* deep learning, *RG* respiratory-gated

^a^ *p*-value of Kruskal–Wallis test

^b^ *p*-value of a post hoc Wilcoxon signed-rank test with the Bonferroni correction

### Lesion assessment

A total of 260 lesions were identified according to the reference standard in 73 of the 120 included magnetic resonance examinations (Table S2).

Reader 1 identified 237 lesions (91.2%) on RG ARMS-T2WI, 246 (94.6%) lesions on RG DL-T2WI and 248 (95.4%) lesions on BH DL-T2WI. No false-positive findings for the three sequences were observed. No significant differences concerning the number of detected lesions between the three sequences for Reader1 (*p* = 0.106). Reader 2 identified 249 (95.8%) lesions on RG DL-T2WI and 243 (93.5%) lesions on BH DL-T2WI, which were significantly higher than RG ARMS-T2WI (232 lesions, 89.2%) (*p* = 0.0137).

There was no statistically significant difference among the three sequences in the measured maximum diameter of detected lesions (*p* = 0.106). Lesion conspicuity scores showed no significant difference for the three sequences (*p* = 0.990; Table 3).

### The relationship between the respiratory curve and image quality

For RG ARMS-T2WI (Table 4), AVGamp peak (ρ = -0.281, *p* = 0.002), SD_amp_ peak (ρ = -0.615, *p* < 0.001), SD_amp_ trough (ρ = -0.341, *p* < *0.001*), AVG_amp_ trigger point (ρ = -0.347, *p* < *0.001*), SD_amp_ trigger point (ρ = -0.545, *p* < 0.001), SD_breath-time_ (ρ = -0.427, *p* < *0.001*), and coefficient of variation of breathing rate (ρ = 0.227, *p* = 0.012) were fairly correlated with overall image quality.

**Table 4:** Spearman’s correlation coefficients between respiratory curve parameters and image quality

	Artifacts	Sharpness of the left liver margin	Sharpness of the right liver margin	Hepatic vessel clarity	CRSL	Overall image quality
RG ARMS-T2WI						
Breathing rate	0.193*	0.120	0.156	0.113	-0.038	0.179
AVGamp peak	-0.275**	-0.096	-0.161	-0.257**	-0.100	-0.281**
SDamp peak	-0.587**	-0.382**	-0.506**	-0.632**	-0.244**	-0.615**
AVGamp trough	-0.075	-0.052	-0.038	-0.137	-0.107	-0.067
SDamp trough	-0.349**	-0.186*	-0.239**	-0.239**	-0.196*	-0.341**
AVGamp trigger point	-0.356**	-0.166	-0.224*	-0.267**	-0.136	-0.347**
SDamp trigger point	-0.534**	-0.399**	-0.432**	-0.567**	-0.145	-0.545**
AVG breath time	-0.181*	-0.120	-0.149	-0.109	0.030	-0.171
SD breath time	-0.455**	-0.285**	-0.326**	-0.426**	-0.026	-0.427**
Coefficient of variation of breathing rate	0.269**	0.129	0.151	0.238**	-0.017	0.227*
RG DL-T2WI						
Breathing rate	0.200*	0.168	0.182*	0.145	-0.109*	0.213
AVGamp peak	-0.310**	-0.150	-0.207*	-0.216*	-0.036	-0.232*
SDamp peak	-0.667**	-0.406**	-0.470**	-0.650**	-0.305**	-0.588**
AVGamp trough	-0.088	-0.136	-0.127	-0.243**	-0.047	-0.145
SDamp trough	-0.423**	-0.425**	-0.357**	-0.398**	-0.149	-0.441**
AVGamp trigger point	-0.360**	-0.258**	-0.312**	-0.279**	-0.099	-0.316**
SDamp trigger point	-0.504**	-0.331**	-0.339**	-0.524**	-0.267**	-0.456**
AVG breath time	-0.210*	-0.203*	-0.227*	-0.174	0.090	-0.241**
SD breath time	-0.409**	-0.264**	-0.305**	-0.372**	-0.112	-0.372**
Coefficient of variation of breathing rate	0.106	0.003	-0.089	0.025	0.094	0.036
BH DL-T2WI						
AVGamp	-0.158	-0.048	-0.117	-0.012	-0.119	-0.178
SDamp	-0.652**	-0.037	-0.099	-0.003	-0.120	-0.221**

The average value and standard deviation of the peaks (AVGamp peak, SDamp peak) and troughs (AVGamp trough, SDamp trough) in the breathing curve, average value (AVGbreath-time) and standard deviation (SDbreath-time) of the time interval between each pair of trigger points, and the average and SD of the respiratory amplitude at the trigger points (AVGamp trigger point and SDamp trigger point). The average value (AVGamp) and standard deviation (SDamp) of the respiratory amplitude

*ARMS* reconstruction with motion suppression, *BH* breathing hold, *CRSL* cardiac motion-related signal loss, *DL* deep learning, *RG* respiratory-gated

* *p* < 0.05; ** *p* < 0.001

For RG DL-T2WI (Table 4), AVG_amp_ peak (ρ = -0.232, *p* = 0.001), SD_amp_ peak (ρ = -0.588, *p* < 0.001), SD_amp_ trough (ρ = -0.441, *p* < 0.001), AVG_amp_ trigger point (ρ = -0.316, *p* < 0.001), SD_amp_ trigger point (ρ = -0.456, *p* < 0.001), AVG breath time (ρ = -0.241, *p* = 0.008), and SD_breath-time_ (ρ = -0.372, *p* < 0.001) were fairly correlated with overall image quality.

For BH DL-T2WI(Table 4), SDamp showed a moderate correlation with artifact (ρ = -0.652, *p* < 0.001) and was fairly correlated with overall image quality (ρ = -0.221, *p* = 0.015).

### Construction and validation of the respiratory signature

**Fig. 4:**
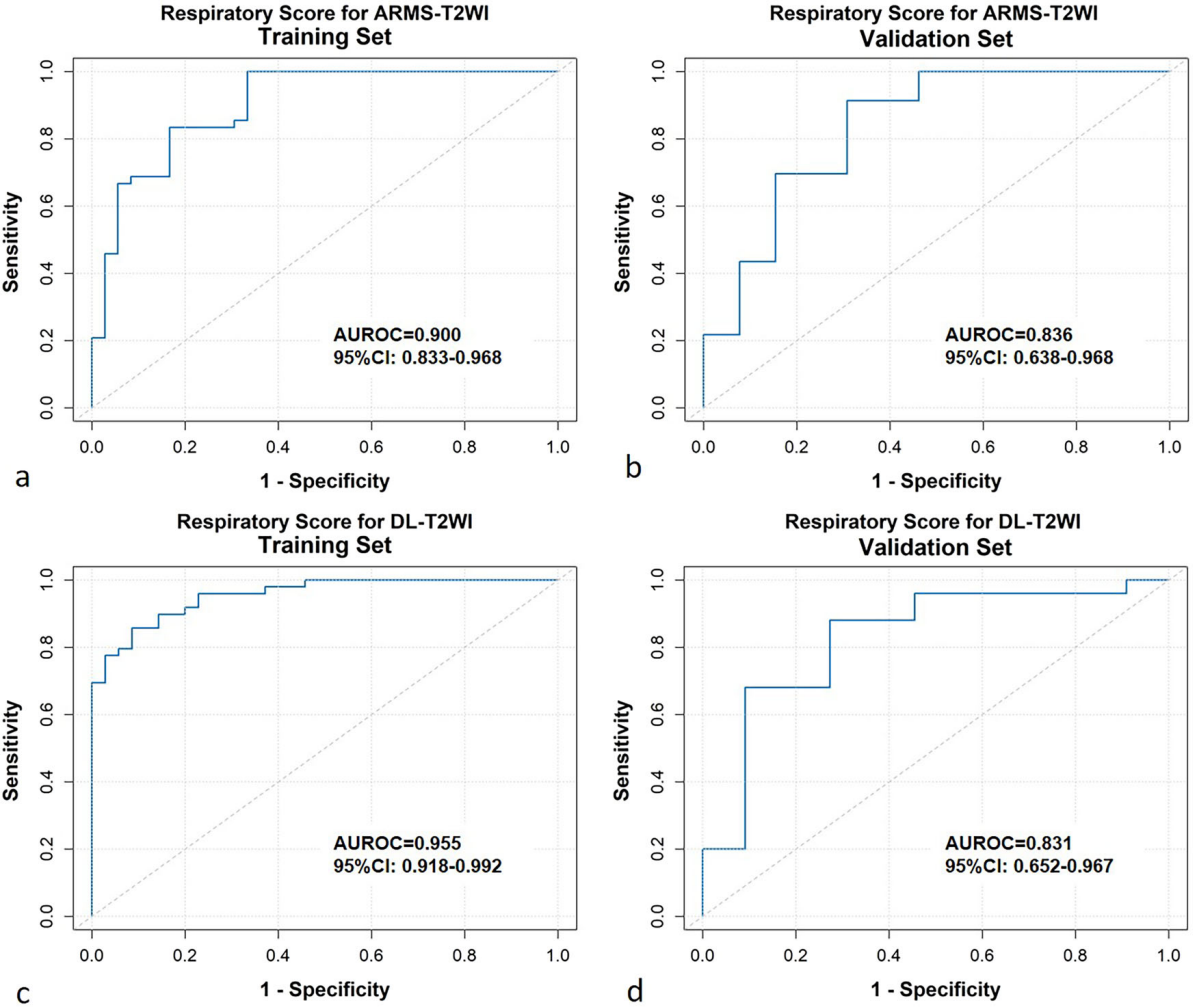
Receiver operating characteristic (ROC) curves illustrating the performance of respiratory scores in predicting image quality for respiratory-gated (RG) ARMS-T2WI (**a**, **b**) and RG deep learning (DL)-T2WI (**c**, **d**) sequences

**Fig. 5:**
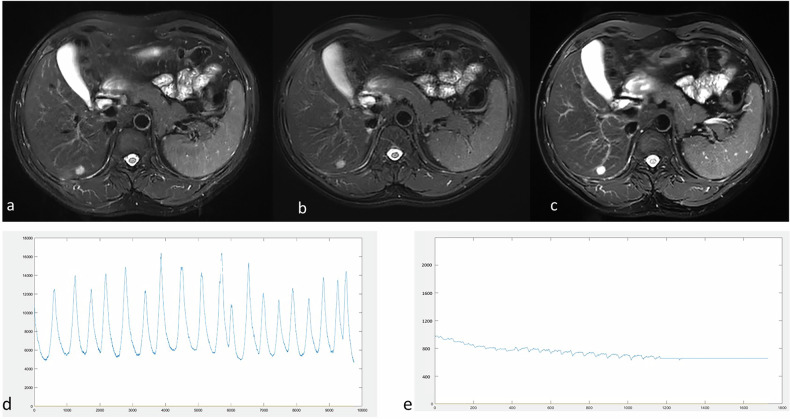
T2WI Images and respiratory curves of a 43-year-old male. **a** Respiratory-gated (RG) ARMS-T2WI (overall image score of 3). **b** RG deep learning (DL)-T2WI (overall image score of 4). **c** Breath-hold DL-T2WI (overall image score of 5). **d** Respiratory curve during free breathing in RG T2WI sequences scanning. The patient showed irregular breathing, with a respiratory score of 0.439 for ARMS-T2WI and 0.663 for RG DL-T2WI. **e** The respiratory curve during breath-hold DL-T2WI scanning shows that the patient maintained a steady breath-hold (indicated by a low SD_amp_)

For RG ARMS-T2WI, the LASSO regression model identified four respiratory parameters most strongly associated with image quality in the training set (Table S3). The optimal penalty parameter was determined as λ.min (λ = 0.041) through 10-fold cross-validation. A respiratory score was then calculated as a linear combination of these selected features, weighted by their respective coefficients. In the training set, the apparent AUROC was 0.900 (95% confidence interval (CI): 0.834‒0.968). Using 1,000 bootstrap resamples, the average optimism was estimated to be 0.034, yielding an optimism-corrected AUROC of 0.867 (95% CI: 0.809‒0.934). In the validation set, the model achieved an AUROC of 0.836, with a bootstrap-derived 95% CI of 0.638‒0.968 (Fig. 4). Calibration plots and decision-curve analysis for both cohorts are presented in Figs. S2 and S3. At a threshold probability of 0.50, the LASSO model provided a standardized net benefit of 0.739 (95% CI: 0.500‒0.920), demonstrating clinically meaningful utility compared with the “treat-all” and “treat-none” strategies. The respiratory score was further dichotomized into a respiratory signature to stratify patients based on their probability of achieving good image quality, using a cutoff of 0.565. Patients with high scores demonstrated significantly better image quality of ARMS-T2WI, compared to those with low scores in both the training (87.0% *versus* 21.1%, *p* < 0.001) and validation sets (83.3% *versus* 25.0%, *p* = 0.002) (Fig. 5). Among patients with low respiratory scores for ARMS-T2WI, 52% (26 of 50) achieved improved image quality with RG DL-T2WI (*p* < 0.001).

For RG DL-T2WI, the LASSO regression model identified seven respiratory parameters most strongly associated with image quality (Table S4). The optimal penalty parameter was determined as λ.min = 0.00239 through 10-fold cross-validation and applied in the final model. In the training set, the apparent AUC was 0.955 (95% CI: 0.918‒0.992). To account for potential overfitting, bootstrap resampling (B = 1,000) was conducted, yielding an average optimism of 0.027 and an optimism-corrected AUROC of 0.942 (95% CI: 0.897‒0.981). The validation set has a bootstrap-corrected mean AUROC of 0.831 (95% CI: 0.652‒0.967) (Fig. 4). To assess clinical utility, decision-curve analysis was performed (Fig. S3). At a representative threshold probability of 0.50, the standardized net benefit reached 0.720 (95% CI: 0.450–0.920), demonstrating added value over default treatment strategies. Together with calibration plots (Fig. S2), these results support the robustness and clinical utility of our model. Patients with high scores (cutoff as 0.596) demonstrated significantly better image quality of RG DL-T2WI, compared to those with low scores in both the training (93.3% *versus* 17.9%, *p* < 0.001) and validation sets (84.5% *versus* 0.3%, *p* = 0.005).

**Fig. 6:**
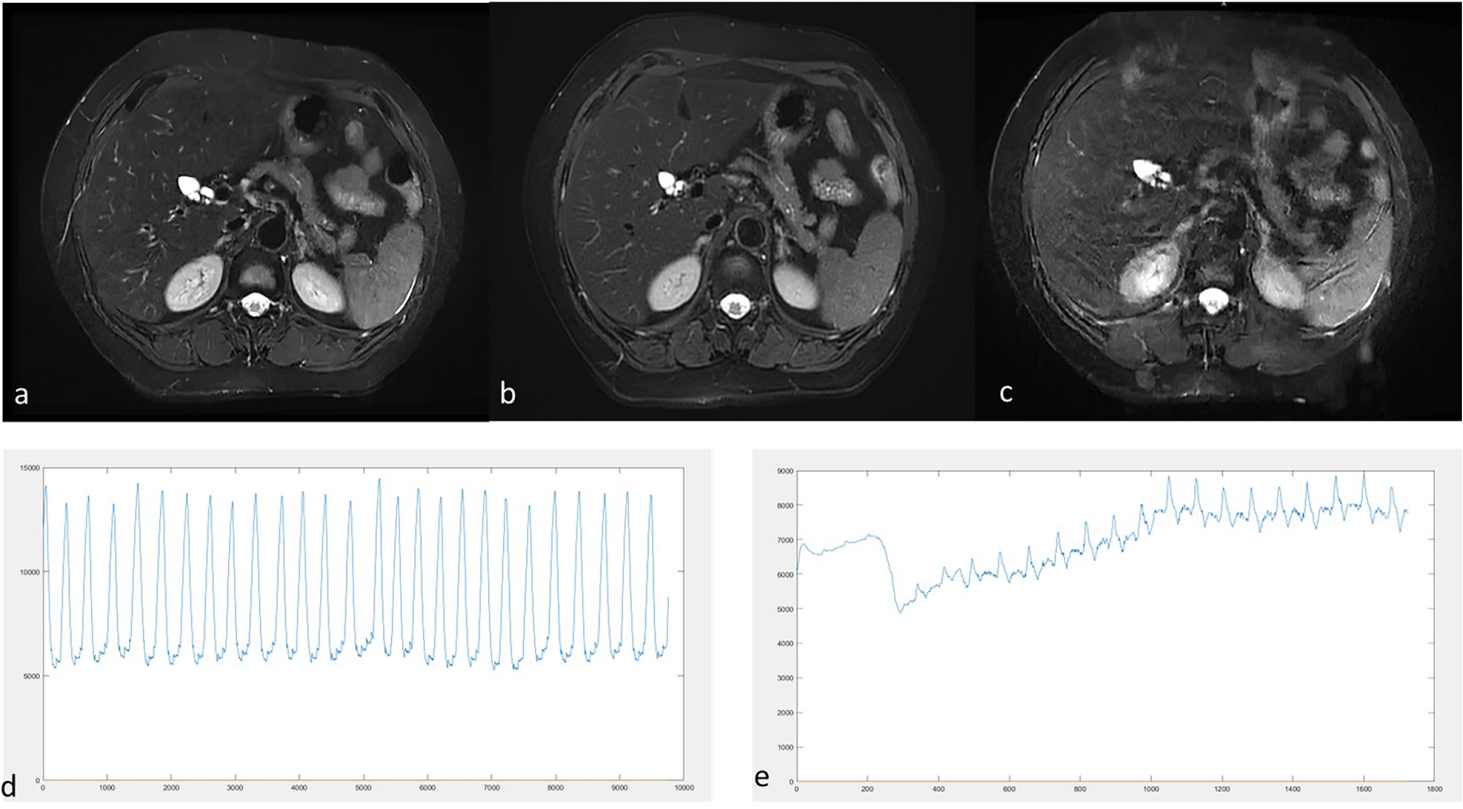
T2WI Images and respiratory curves of a 56-year-old male. **a** Respiratory-gated (RG)ARMS-T2WI (overall image score of 4). **b** RG deep learning (DL)-T2WI (overall image score of 5). **c** Breath-hold DL-T2WI (overall image score of 2). **d** Respiratory curve during free breathing in RG T2WI sequences scanning. The patient showed regular breathing and relatively fast breathing rhythm, with a respiratory score of 0.791 for ARMS-T2WI and 0.992 for RG DL-T2WI. **e** The respiratory curve during breath-hold DL-T2WI scanning shows that the patient was unable to maintain the breath-hold (indicated by a high SD_amp_)

For BH DL-T2WI (Table S5), SD_amp_ was identified as an independent factor of good image quality in the training set (odds ratio = 0.517, 95% Cl: 0.277‒0.893, *p* = 0.025) and validation set (odds ratio = 0.297, 95% Cl: 0.066‒0.826, *p* = 0.049). The AUC values of SD*amp* for predicting image quality of BH DL-T2WI in the training and validation sets were 0.626 (95% CI: 0.501‒0.750) and 0.667 (95% CI: 0.489‒0.845), respectively. Among patients with high SD_amp_ values (cutoff of 0.364), only 5 out of 35 achieved good overall image quality with the BH DL-T2WI sequence, whereas 24 patients attained good overall image quality with the RG DL-T2WI sequence (*p* < 0.001) (Fig. 6).

### Inter-reader agreement

Inter-reader agreement between the three readers for all subjective assessments of image quality was good to almost perfect, with values between 0.850 and 0.973 (Tables S6–S8).

The inter-reader agreement was excellent for the measured maximum diameter of detected lesions for the three sequences (RG ARMS-T2WI: 24.59 ± 24.70 *versus* 24.72 ± 24.53 mm, ICC = 0.999; RG DL-T2WI: 24.63 ± 24.86 *versus* 24.88 ± 24.80 mm, ICC = 0.996; BH DL-T2WI: 24.63 ± 24.68 *versus* 24.51 ± 24.72 mm, ICC = 0.994).

## Discussion

In this study, we demonstrated that both RG and BH DL-T2WI achieve overall imaging quality and diagnostic performance comparable to radial k-space sampling–based T2WI, while significantly reducing scan time. Additionally, we observed that the respiratory characteristics of patients variably influenced image quality across different T2WI sequences. This implies that respiratory parameters could effectively predict image quality and serve as critical indicators for developing personalized imaging liver MRI workflows.

While liver T2WI sequences usually require extended scan times, in this study, the average scan time for the respiratory-gated ARMS-T2WI sequence was only 3‒4 min, with some patients needing up to 6.5 min. The prolonged scan times increase the likelihood of patient movement and cause changes in their breathing patterns, which further extend the overall scanning duration [13]. In contrast, the breath-hold DL-T2WI sequence can capture all slices in just 17 s during a single breath-hold. However, according to our results, only 43.3% of cases achieved excellent overall image quality for BH DL-T2WI, which was significantly lower than RG DL-T2WI and RG ARMS-T2WI sequences. For postoperative, frail, or elderly patients, 17 s may still be too long. In such cases, the RG DL-T2WI sequence is preferable. This sequence allows data collection during free breathing, with an average scan time of 32 s, typically completed within 6 to 10 breathing cycles.

Respiratory motion is a key factor affecting image quality, but few studies have detailed the relationship between individual breathing characteristics and image quality. Park et al conducted a subjective evaluation of respiratory curve characteristics and found that patients’ breath-holding capacity significantly impacts hepatic arterial phase image quality [32]. Similarly, He et al subjectively assessed breath regularity through respiratory waves and found that, for patients with irregular breathing, breath-hold magnetic resonance cholangiopancreatography demonstrated superior overall imaging quality and diagnostic performance compared to RG acquisition [33]. However, assessing patients’ respiratory characteristics based on respiratory rhythm and amplitude requires substantial clinical experience and is highly influenced by subjective factors. To address this, Wang et al quantified patients’ respiratory curve data into specific parameters, effectively demonstrating the relationship between patients’ respiratory characteristics and magnetic resonance cholangiopancreatography image quality [15].

However, the impact of patients’ respiratory characteristics on liver T2WI imaging has not been thoroughly studied. In recent years, T2WI imaging has utilized sophisticated k-space sampling strategies and deep learning-based reconstruction, alongside respiratory- or navigator-triggered acquisitions, to reduce respiratory motion artifacts [13, 34–36]. The impact of patients’ respiratory characteristics on image quality may vary across different imaging techniques. Understanding the influence of respiratory characteristics on the image quality of various imaging sequences can facilitate the development of a personalized workflow throughout the imaging process.

We also noted that BH DL-T2WI showed significantly lower scores of the sharpness of intrahepatic vessel margins and overall image quality compared with respiratory gating sequences. This is partly because a significant number of patients are unable to fully hold their breath and are unaware of the air leakage from their nose, even after breathing training. According to a previous study, the incidence of breath-holding difficulty during the hepatic arterial phase was 23.4–34.6% [14, 32]. In our study, breath-holding difficulties were reflected by slight fluctuations at the end of the breath-hold on the respiratory curve, reflected by the SD_amp_ value. Identified as an independent determinant of image quality for BH DL-T2WI, SD_amp_ was used to categorize patients into low and high-value groups. The overall image quality of BH DL-T2WI in the high SD_amp_ group was inferior to that of the RG DL and ARMS-T2WI images. Hence, although the BH DL-T2WI sequence reduces artifacts and enables rapid scanning, it is not suitable for all patients.

This study has several limitations. First, this single-institutional study involved a relatively small number of patients with limited variants of respiratory characteristics, leading to a selection bias potential. Our results require validation through multicenter studies and large cohort analyses. Second, this study solely assessed the lesion conspicuity of the three sequences, with reference standards derived from a combination of multimodal imaging findings. Further research is needed to determine the clinical significance and diagnostic efficacy for specific diseases. Third, although the three T2WI sequences were acquired in random order, the additional scan time likely increased patient fatigue and discomfort, which subsequently affected image quality. Fourth, we used a commercial inline deep learning image reconstruction; however, proprietary model specifications and weights are not publicly available, which limits code-level reproducibility and cross-vendor generalizability. Accordingly, our conclusions are based on reader-assessed image quality, conspicuity, and lesion detection outcomes. External multicenter and cross-vendor validation studies are therefore still warranted. Fifth, because we used vendor-validated, site-optimized 3-T protocols, acquisition parameters were not identical across sequences; we matched slice thickness, interslice gap, and echo time to limit confounding, but residual effects from unmatched settings may persist.

In conclusion, both RG and BH DL-T2WI sequences offer reliable image quality with significantly reduced scan times compared to radial acquisition T2WI. Variability in patient respiratory characteristics influences image quality across sequences, underscoring their potential to inform personalized imaging protocols in clinical practice.

## Supplementary information

Supplementary information: **Table S1** Details of the 5-point Likert-type scale in the assessment for image quality.**Table S2** Distribution of Lesion Types. **Table S3** Respiratory parameters and corresponding coefficients identified by LASSO regression for predicting image quality of respiratory-gated ARMS-T2WI. **Table S4** Respiratory parameters and corresponding coefficients identified by LASSO regression for predicting image quality of respiratory-gated DL-T2WI. **Table S5** Multivariate logistic regression analysis of curve parameters on overall image quality of breath-hold deep learning (DL)-T2WI sequence. **Table S6** The inter-reader agreement of subjective assessment of image quality for the respiratory-gated (RG) ARMS-T2WI sequence. **Table S7** The inter-reader agreement of subjective assessment of image quality for the respiratory-gated (RG) deep learning (DL)-T2WI sequence. **Table S8** The inter-reader agreement of subjective assessment of image quality for the breath-hold deep learning (DL)-T2WI sequence. **Fig. S1**: Representative examples of ambiguous/non-lesion exclusions. (a) Motion ghosting on T2WI mimicking a focal hyperintense nodule, with no corresponding lesion on contrast-enhanced MRI. (b) Peribiliary cysts appearing as parenchymal nodules on T2WI, not visible on other sequences. (c) Vessel-related partial-volume hyperintensity at the hepatic vein boundary on T2WI, correctly identified by contrast-enhanced images. (d) Ill-defined subcapsular edema with T2 hyperintensity, without enhancement on dynamic imaging. **Fig. S2**: Calibration plots of respiratory-gated (RG) ARMS-T2WI and RG deep learning (DL)-T2WI-based LASSO respiratory models for predicting image quality in training and validation cohorts. The plots illustrate the agreement between predicted and observed probabilities of image quality, with the diagonal line representing perfect calibration and the solid lines showing model performance. **Fig. S3**: Decision-curve analysis (DCA) of respiratory-gated (RG) ARMS-T2WI and RG deep learning (DL)-T2WI-based LASSO respiratory models for predicting image quality in training and validation cohorts. The curves demonstrate the net clinical benefit across a range of threshold probabilities, indicating the added value of the models compared with the default strategies of assuming all or no images have adequate quality.

## Data Availability

The original data and code used in this study are available from the corresponding author upon reasonable request.

## Abbreviations

ARMS: Acquisition and reconstruction with motion suppression
AUROC: Area under the receiver operating characteristic curve
AVG_amp_: Average value of the respiratory amplitude
AVG_breath-time_: Average of the time intervals between each pair of trigger points
BH: Breath-hold
CI: Confidence interval
CR: Contrast ratio
CRSL: Cardiac motion-related signal loss
DL: Deep learning
FSE: Fast spin-echo
LASSO: Absolute shrinkage and selection operator
MRI: Magnetic resonance imaging
ROI: Region of interest
SD_amp_: Standard deviation of the respiratory amplitude
SD_breath-time_: Standard deviation of the time intervals between each pair of trigger points
SNR: Signal-to-noise ratio
