# Supplementary material for: Comparison of respiratory-gated and breath‑hold accelerated T2-weighted sequences for liver MRI with deep learning reconstruction
Source: Eur Radiol Exp. 2026 Feb 23;10:13. doi: 10.1186/s41747-026-00679-1 (PMC12929759; doi:10.1186/s41747-026-00679-1)
Supplement: Supplementary file 1 — Supplementary information: Table S1 Details of the 5-point Likert-type scale in the assessment for image quality.Table S2 Distribution of Lesion Types. Table S3 Respiratory parameters and corresponding coefficients identified by LASSO regression for predicting image quality of respiratory-gated ARMS-T2WI. Table S4 Respiratory parameters and corresponding coefficients identified by LASSO regression for predicting image quality of respiratory-gated DL-T2WI. Table S5 Multivariate logistic regression analysis of curve parameters on overall image quality of breath-hold deep learning (DL)-T2WI sequence. Table S6 The inter-reader agreement of subjective assessment of image quality for the respiratory-gated (RG) ARMS-T2WI sequence. Table S7 The inter-reader agreement of subjective assessment of image quality for the respiratory-gated (RG) deep learning (DL)-T2WI sequence. Table S8 The inter-reader agreement of subjective assessment of image quality for the breath-hold deep learning (DL)-T2WI sequence. Fig. S1: Representative examples of ambiguous/non-lesion exclusions. (a) Motion ghosting on T2WI mimicking a focal hyperintense nodule, with no corresponding lesion on contrast-enhanced MRI. (b) Peribiliary cysts appearing as parenchymal nodules on T2WI, not visible on other sequences. (c) Vessel-related partial-volume hyperintensity at the hepatic vein boundary on T2WI, correctly identified by contrast-enhanced images. (d) Ill-defined subcapsular edema with T2 hyperintensity, without enhancement on dynamic imaging. Fig. S2: Calibration plots of respiratory-gated (RG) ARMS-T2WI and RG deep learning (DL)-T2WI-based LASSO respiratory models for predicting image quality in training and validation cohorts. The plots illustrate the agreement between predicted and observed probabilities of image quality, with the diagonal line representing perfect calibration and the solid lines showing model performance. Fig. S3: Decision-curve analysis (DCA) of respiratory-gated (RG) ARMS-T2WI and [file 41747_2026_679_MOESM1_ESM.pdf]

Comparison of respiratory-gated and breath-hold accelerated T2-weighted sequences for liver MRI with deep learning reconstruction

ELECTRONIC SUPPLEMENTARY MATERIAL

Table S1 Details of the 5-point likert-type scale in the assessment for image quality.

	1	2	3	4	5
Artifacts	severe artifacts rendering the images with nondiagnostic	severe artifacts with insufficient diagnostic confidence	Moderate artifacts with some uncertainty in diagnosis	Minor artifacts without loss of diagnostic value	No detectable artifact
Sharpness of liver margin	No visible boundary	indistinct boundary	blurred boundary	fairly clear boundary	clear boundary
Hepatic vessel clarity	almost invisible	extremely blurred	moderately blurred	slightly blurred	not blurred
CRSL	nondiagnostic	significant loss affecting diagnosis	partial loss with diagnostic interference	minor loss without diagnostic impact	excellent, no signal loss
Lesion conspicuity	not visible	partially faintly visible	partially visible	visible with blurred edges	clearly visible with distinct edges
Overall image quality	nondiagnostic	poor quality without loss of diagnostic value	fair quality with some uncertainty in diagnosis	good quality without loss of diagnostic value	excellent quality

CRSL, cardiac motion–related signal loss.

Table S2 Distribution of Lesion Types.

Verification method	Lesion type	0–5 mm	5–10 mm	>10 mm	Total
Histopathology	Tumor	3	4	39	46
	FNH	0	0	1	1
Follow-up imaging	Cyst	74	27	30	131
	Hemangioma	6	0	10	16
	Tumor	3	5	20	28
Multiparametric MRI	Cyst	4	3	16	23
	Hemangioma	0	3	11	14
	Tumor	0	0	1	1
Total	All	90	42	128	260

Table S3 Respiratory parameters and corresponding coefficients identified by LASSO regression for predicting image quality of respiratory-gated ARMS-T2WI.

Respiratory parameters	Coefficients
AVGamp trigger point	-0.084
Sdamp trigger point	-0.037
SDamp peak	-0.116
Coefficient_of_variation_of_breathing_rate	0.052

The standard deviation of the peaks (SDamp peak) in the breathing curve, and the average and SD of the respiratory amplitude at the trigger points (AVGamp trigger point and SDamp trigger point).

Table S4 Respiratory parameters and corresponding coefficients identified by LASSO regression for predicting image quality of respiratory-gated DL-T2WI.

Respiratory parameters	coefficients
Breathing rate	-0.151
AVG breath time	-0.173
AVGamp peak	-0.023
SDamp peak	-0.068
AVGamp trigger point	-0.027
SDamp trigger point	-0.103
Coefficient of variation of breathing rate	0.219

The average value and standard deviation of the peaks (AVGamp peak, SDamp peak) in the breathing curve, average value (AVG breath-time) of the time interval between each pair of trigger points, and the average and SD of the respiratory amplitude at the trigger points (AVGamp trigger point and SDamp trigger point).

Table S5 Multivariate logistic regression analysis of curve parameters on overall image quality of breath-hold deep learning (DL)-T2WI sequence.

Parameters	Training set			Validation set		
	OR	95%CI	*p*	OR	95%CI	*p*
Sex	0.475	0.165-1.311	0.156	2.165	0.412-12.987	0.368
Age	0.984	0.947-1.021	0.405	1.039	0.974-1.118	0.264
AVGamp	0.611	0.340-1.032	0.077	0.691	0.188-1.981	0.525
SDamp	0.517	0.277-0.893	**0.025**	0.297	0.066-0.826	**0.049**

AVGamp, The average value and standard deviation of the respiratory amplitude; SDamp, The sta -ndard deviation of the respiratory amplitude.
BH, Breath-hold; DL, Deep learning; OR, Odds ratio; CI, Confidence interval; RG, Respiratory-gat ed.

Table S6 The inter-reader agreement of subjective assessment of image quality for the respiratory-gated (RG) ARMS-T2WI sequence.

	Reader1	Reader2	Reader3	ICC
Artifacts	3.51±1.13	3.5±1.17	3.49±1.16	0.973
Sharpness of left liver margin	4.23±0.67	4.21±0.72	4.23±0.69	0.908
Sharpness of right liver margin	4.06±0.71	4.06±0.74	4.06±0.74	0.948
Hepatic vessel clarity	3.86±1.03	3.88±1.01	3.86±1.01	0.968
CRSL	4.47±0.71	4.46±0.71	4.46±0.71	0.950
Overall image quality	3.47±1.03	3.48±1.06	3.48±1.06	0.965
Lesion conspicuity	4.16±0.92	4.19±0.92	4.19±0.92	0.930

ARMS, Reconstruction with motion suppression; CRSL, Cardiac motion-related signal loss; ICC, Intra-class correlation coefficient; RG, Respiratory-gated.

Table S7 The inter-reader agreement of subjective assessment of image quality for the respiratory-gated (RG) deep learning (DL)-T2WI sequence.

	Reader1	Reader2	Reader3	ICC
Artifacts	4.05±0.95	4.04±0.95	4.04±0.95	0.957
Sharpness of left liver margin	4.27±0.66	4.27±0.66	4.27±0.66	0.904
Sharpness of right liver margin	4.07±0.69	4.08±0.69	4.08±0.69	0.901
Hepatic vessel clarity	4.04±0.87	4.03±0.87	4.03±0.87	0.941
CRSL	3.79±0.83	3.78±0.82	3.78±0.82	0.926
Overall image quality	4.0±0.87	4.02±0.88	4.02±0.88	0.913
Lesion conspicuity	4.34±0.85	4.44±0.75	4.44±0.75	0.850

CRSL, Cardiac motion-related signal loss; DL, Deep learning; ICC, Intra-class correlation coefficient; RG, Respiratory-gated.

Table S8 The inter-reader agreement of subjective assessment of image quality for the breath-hold deep learning (DL)-T2WI sequence.

	Reader1	Reader2	Reader3	ICC
Artifacts	3.97±0.76	3.98±0.78	3.98±0.78	0.935
Sharpness of left liver margin	4.21±0.58	4.21±0.61	4.21±0.61	0.907
Sharpness of right liver margin	3.98±0.65	4.0±0.66	4.0±0.66	0.910
Hepatic vessel clarity	3.57±0.74	3.58±0.76	3.58±0.76	0.946
CRSL	3.53±0.73	3.53±0.74	3.53±0.74	0.929
Overall image quality	4.34±0.75	4.33±0.75	4.33±0.75	0.936
Lesion conspicuity	4.32±0.84	4.37±0.77	4.37±0.78	0.940

BH, Breath-hold; CRSL, Cardiac motion-related signal loss; DL, Deep learning; ICC, Intra-class correlation coefficient.

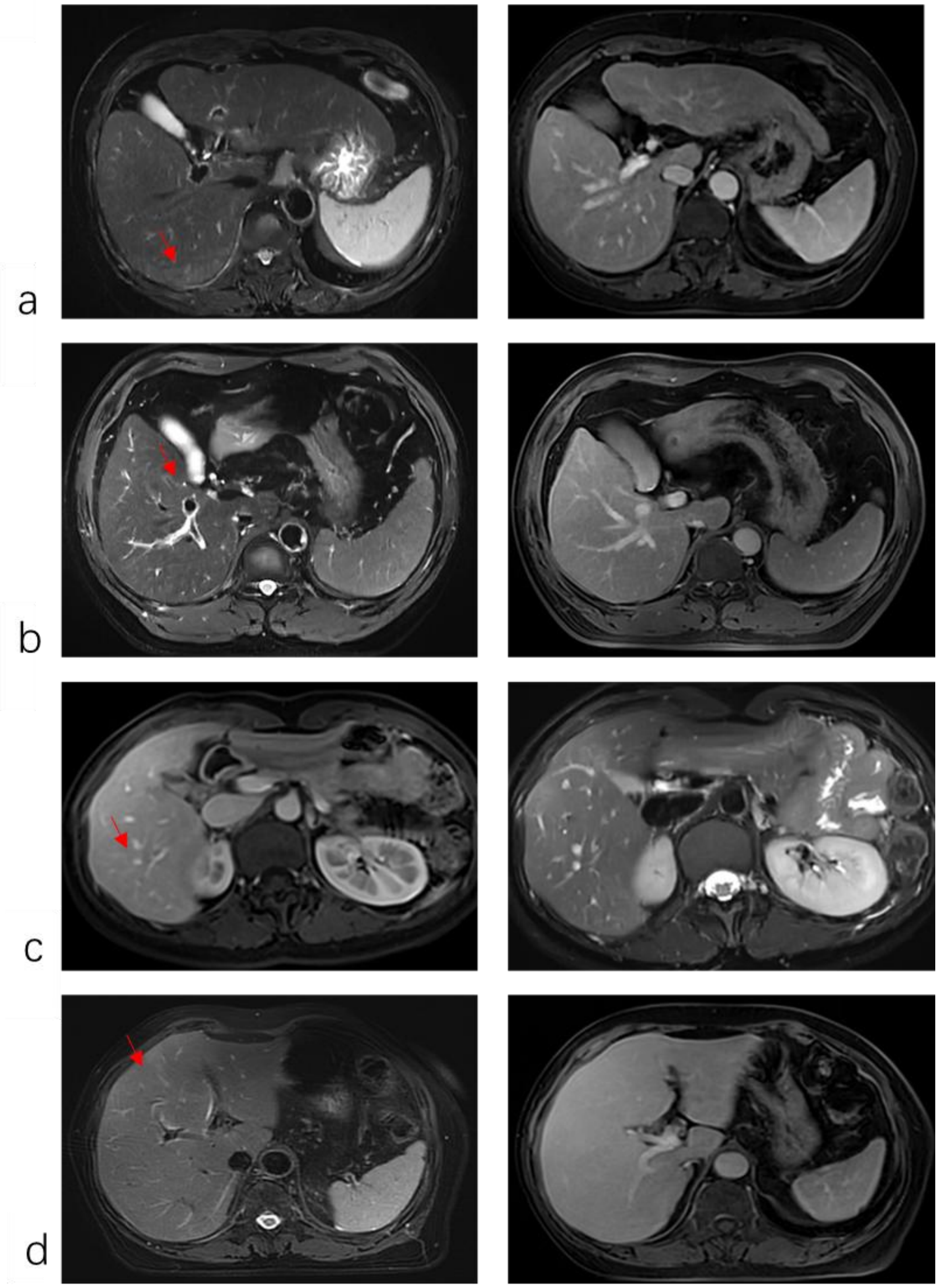

Fig. S1: Representative examples of ambiguous/non-lesion exclusions. (a) Motion ghosting on T2WI mimicking a focal hyperintense nodule, with no corresponding lesion on contrast-enhanced MRI. (b) Peribiliary cysts appearing as parenchymal nodules on T2WI, not visible on other sequences. (c) Vessel-related partial-volume hyperintensity at the hepatic vein boundary on T2WI, correctly identified by contrast-enhanced images. (d) Ill-defined subcapsular edema with T2 hyperintensity, without enhancement on dynamic imaging.

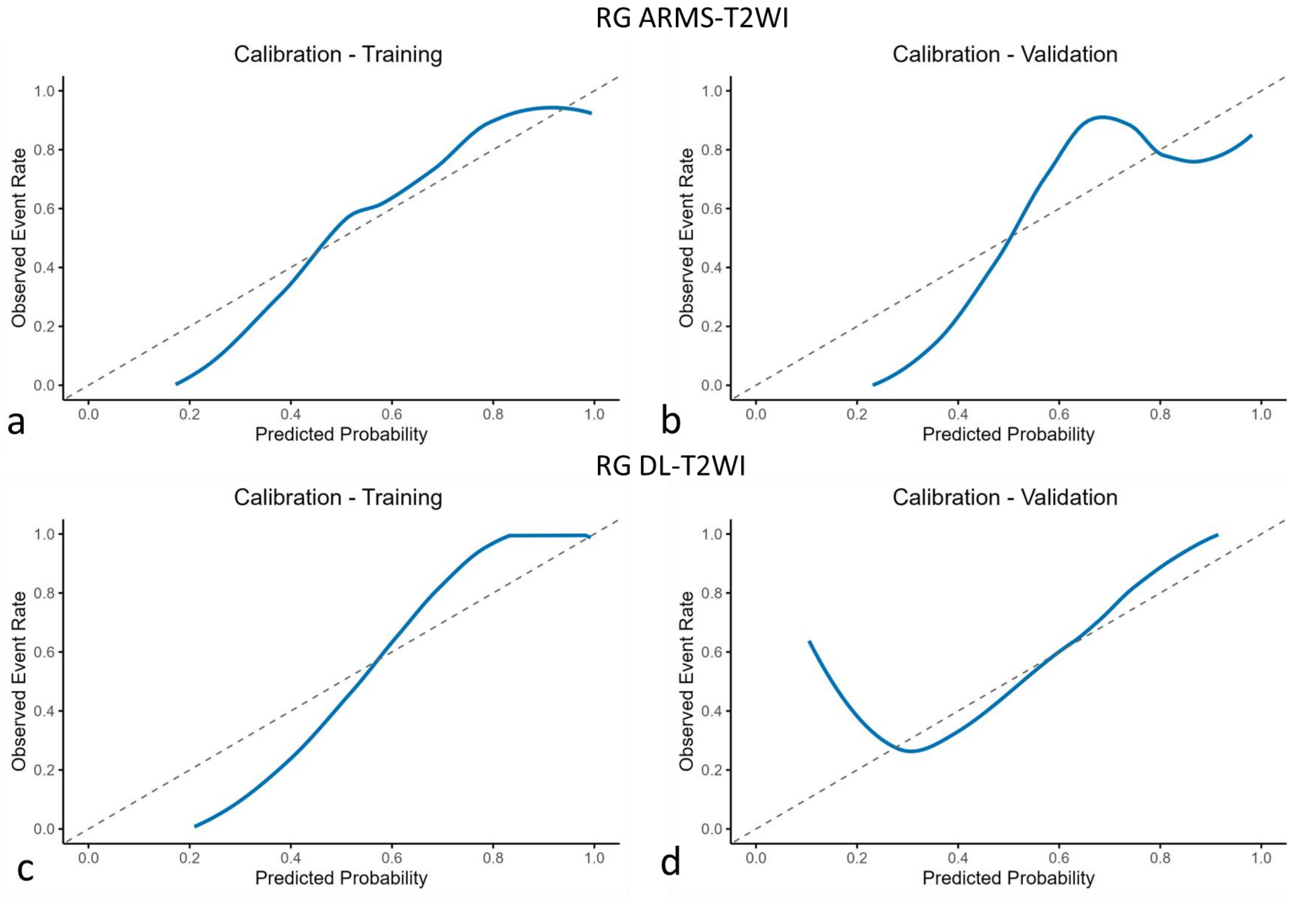

Fig. S2: Calibration plots of respiratory-gated (RG) ARMS-T2WI and RG deep learning (DL)-T2WI-based LASSO respiratory models for predicting image quality in training and validation cohorts. The plots illustrate the agreement between predicted and observed probabilities of image quality, with the diagonal line representing perfect calibration and the solid lines showing model performance.

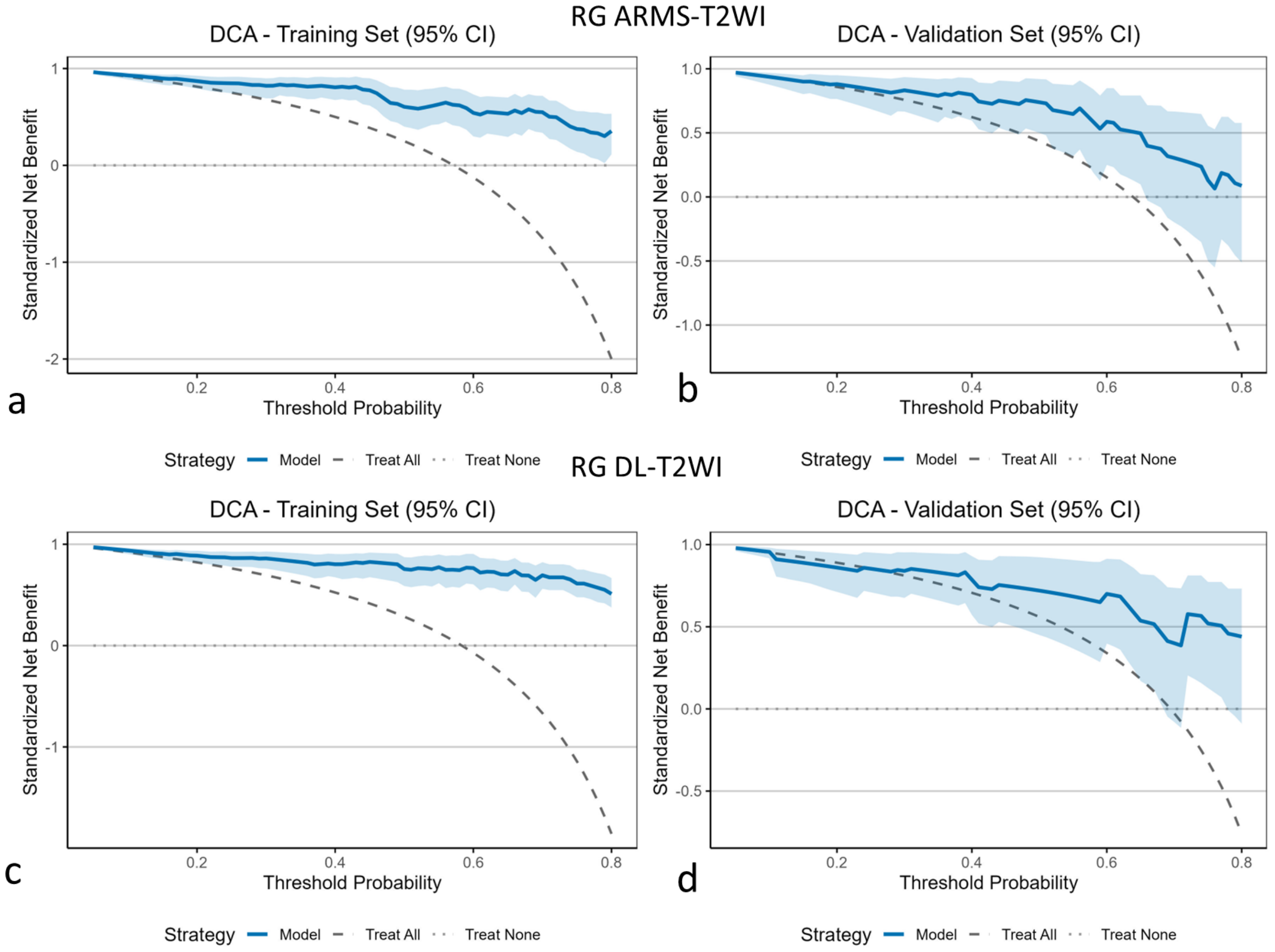

Fig. S3: Decision curve analysis (DCA) of respiratory-gated (RG) ARMS-T2WI and RG deep learning (DL) -T2WI-based LASSO respiratory models for predicting image quality in training and validation cohorts. The curves demonstrate the net clinical benefit across a range of threshold probabilities, indicating the added value of the models compared with the default strategies of assuming all or no images have adequate quality.
